# End-to-End Deep-Learning-Based Diagnosis of Benign and Malignant Orbital Tumors on Computed Tomography Images

**DOI:** 10.3390/jpm13020204

**Published:** 2023-01-23

**Authors:** Ji Shao, Jiazhu Zhu, Kai Jin, Xiaojun Guan, Tianming Jian, Ying Xue, Changjun Wang, Xiaojun Xu, Fengyuan Sun, Ke Si, Wei Gong, Juan Ye

**Affiliations:** 1 Department of Ophthalmology, The Second Affiliated Hospital of Zhejiang University, School of Medicine, Hangzhou 310009, China; 2 Center for Neuroscience and Department of Neurobiology of the Second Affiliated Hospital, State Key Laboratory of Modern Optical Instrumentation, Zhejiang University School of Medicine, Hangzhou 310027, China; 3 Department of Radiology, The Second Affiliated Hospital of Zhejiang University, School of Medicine, Hangzhou 310009, China; 4 Tianjin International Joint Research and Development Centre of Ophthalmology and Vision Science, Eye Institute and School of Optometry, Tianjin Medical University Eye Hospital, Tianjin Medical University, Tianjin 300203, China

**Keywords:** hemangioma, lymphoma, deep learning, segmentation, classification

## Abstract

Determining the nature of orbital tumors is challenging for current imaging interpretation methods, which hinders timely treatment. This study aimed to propose an end-to-end deep learning system to automatically diagnose orbital tumors. A multi-center dataset of 602 non-contrast-enhanced computed tomography (CT) images were prepared. After image annotation and preprocessing, the CT images were used to train and test the deep learning (DL) model for the following two stages: orbital tumor segmentation and classification. The performance on the testing set was compared with the assessment of three ophthalmologists. For tumor segmentation, the model achieved a satisfactory performance, with an average dice similarity coefficient of 0.89. The classification model had an accuracy of 86.96%, a sensitivity of 80.00%, and a specificity of 94.12%. The area under the receiver operating characteristics curve (AUC) of the 10-fold cross-validation ranged from 0.8439 to 0.9546. There was no significant difference on diagnostic performance of the DL-based system and three ophthalmologists (*p* > 0.05). The proposed end-to-end deep learning system could deliver accurate segmentation and diagnosis of orbital tumors based on noninvasive CT images. Its effectiveness and independence from human interaction allow the potential for tumor screening in the orbit and other parts of the body.

## 1. Introduction

Early diagnosis of orbital tumors is critical for timely intervention, appropriate treatment selection, and prognosis prediction. Different orbital tumors have varying treatment protocols owing to their distinct cellular origins. Hemangioma is the most common primary benign orbital tumor, accounting for 6% of all orbital lesions [1]. It is characterized by slow growth [2]. Immediate surgical excision is only recommended in case of evident vision deterioration, while conservative treatment is a valid option for patients in the absence of vision deficits [3,4,5]. Contrastingly, radiotherapy is the first-line treatment for malignant lymphomas [6,7], which are the most common malignant neoplasms in adults [1,8]. The risk of malignant tumor metastases and recurrence increases without optimal clinical intervention, appropriate therapy protocol, and follow-up frequency. Therefore, the early diagnosis and timely treatment of orbital tumors are of crucial importance.

Computed tomography (CT) and magnetic resonance imaging (MRI) are the imaging modalities of choice for detecting orbital tumors [9,10]. Previously, ultrasound was a rapid, inexpensive, and non-invasive medical technology, but it is not the first choice for orbital tumor classification due to its limited value of size detection, location, and relationship to the surrounding structures [11]. CT and MRI are capable of providing images inside the orbit via radio waves and magnetic fields [12,13]. These techniques narrow the differential diagnosis and allow disease staging by assessing the tumor location, homogeneity, margin, signal intensity, and relationship with adjacent structures [10,14,15]. Compared with CT, MRI has an advantage in higher soft tissue resolution, but the application of orbital tumor screening is limited because of the expensive cost and long exam time. Contrast-enhanced CT and MRI could visualize blood flow in various types of orbital tumors, which is beneficial for diagnosing malignant and benign orbital tumors. However, contrast-enhanced CT and MRI are invasive technologies and cannot be performed on patients with contraindications, which include contrast agent allergy, renal insufficiency, claustrophobia, etc. [16]. All of these factors preclude the possibility of contrast-enhanced CT and MRI being well-suited orbital tumor screening tools. Non-contrast-enhanced CT is widely used for detecting orbital tumors, but the imaging findings often overlap and hence pose a diagnostic challenge [17].

Artificial intelligence (AI), a branch of computer science, represents the techniques that enable computers to simulate and extend human intelligence. Machine learning (ML) is a mainstream of AI technology, which is capable of automatically improving computer algorithms by analyzing the features of input rather than following explicit program instructions [18,19]. Conventional ML and deep learning (DL) are two variations of ML [20,21]. With the excellent ability to analyze high-level features, interpreting medical images based on DL has gradually become the research hotspot. 

In recent years, DL has been paid increasing attention in the field of ophthalmology, and many DL-based models have been proposed to assist in the automatic diagnosis, grading, and prognosis prediction of various ocular diseases [22,23,24]. With the need for orbital image analysis skyrocketing, more and more DL networks have been applied to detect and segment intricate anatomical structures of the orbit [25,26]. The capability of achieving pixel-level classification on CT images using DL-based algorithms implies the potential for precise segmentation of orbital tumors. Previous studies had attempted to distinguish ocular adnexal lymphoma from idiopathic orbital inflammation on MRI images and reached diagnostic performance superior to the radiology resident [27,28]. DL-based models have also displayed outstanding performance for automatic tumor classification in medical imaging of multiple organs, such as the lung [29,30], brain [31], and breast [32]. Taken together, we hypothesized that DL-based algorithms had the efficacy of high-precision segmentation and classification of orbital tumors on CT images.

In this study, we retrospectively collected 602 non-contrast-enhanced CT images of 64 patients with orbital tumors (using cavernous hemangiomas and lymphomas as representatives of benign and malignant tumors) from two independent hospitals. An end-to-end DL-based model was established to automatically segment orbital tumors and distinguish malignant orbital tumors from benign ones.

## 2. Materials and Methods

### 2.1. Patients

This retrospective study was reviewed and approved by the institutional review board. All the patients enrolled in this study were recruited from two independent institutions: the Second Affiliated Hospital of Zhejiang University, from February 2009 to June 2020, and Tianjin Medical University Eye Hospital, from December 2017 to May 2018. The inclusion criteria were as follows: (1) histopathological diagnosis of hemangioma or lymphoma based on the excised tissues from the orbital lesions and (2) patients with non–contrast-enhanced CT images before surgery. The exclusion criteria were: (1) patients with unclear histopathological results; (2) poor CT image quality or without CT images in axial view; and (3) history of eye or orbital surgery, trauma, radiation treatment, or other orbital therapy.

### 2.2. CT Image Acquisition and Annotation

Non-contrast-enhanced CT scans (Sensation 16, Somatom perspective, Somatom Definition AS, and Somatom Definition Flash, Siemens Healthcare, Erlangen, Germany; Optima CT540, LightSpeed RT16, and ACTs, GE Healthcare, Milwaukee, USA) were performed on all patients with benign and malignant orbital tumors before surgical excision. The scanning parameters were as follows: 120–130 kVp, 180–300 mAs, 0.94 spiral pitch, and 1.0–4.0 mm slice thickness.

In all CT images, those showing orbital masses in the axial view were selected. A certified ophthalmologist with 2 years of experience used the ITK-SNAP software (version 3.6.0, www.itksnap.org, accessed on 21 January 2023) to manually annotate the ROI in each image. All labels were saved as TIFF images.

### 2.3. Preprocessing and Network Architecture

**Figure 1:**
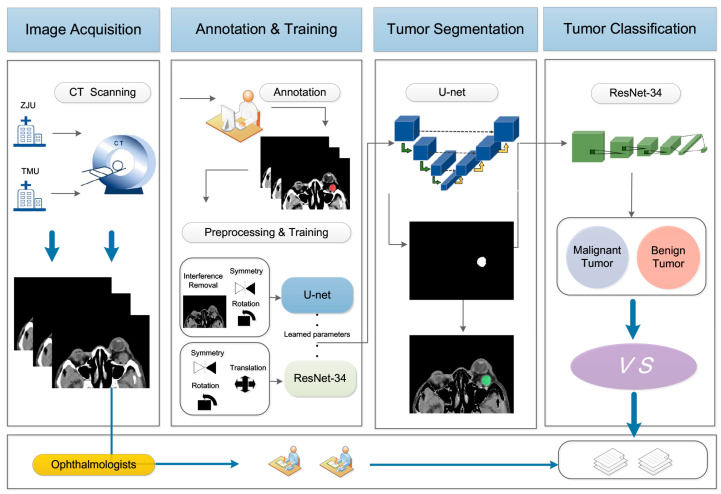
Workflow of the proposed method; CT, computed tomography.

In this study, a total of 602 non-contrast-enhanced CT images from 64 patients were included, which were randomly divided into a training set with 482 images, a validation set with 51 images, and a testing set with 69 images. An end-to-end DL-based system composed of U-Net and ResNet-34 was established to detect and classify orbital tumors. We trained these two sub-networks independently and finally tested the entire system. Figure 1 illustrates the workflow of this study.

#### 2.3.1. Preprocessing of the CT Images

In the preprocessing step, the original CT images in the format of DICOM were converted into 512 × 512 grayscale images in the format of TIFF. To avoid misidentifying non-tumor tissue with high signals as orbital tumors, the grayscale values of all pixels with more than 200 were reset to zero. Then, to speed up the training process, the input images were resized to 256 × 256 resolution. The data augmentation technique was applied to solve the overfitting caused by the small dataset. Symmetry and rotation transformation (with a maximum rotation angle of 10°) were adopted in the U-Net training set. In the ResNet-34 training set, a translational transformation (horizontal and vertical) was used.

#### 2.3.2. Segmentation Network

The automatic segmentation network was trained with U-Net as the backbone. U-Net is one of the most popular network structures in medical image segmentation, which shows advanced performance in the segmentation of small targets [33]. In this study, we used the non-contrast-enhanced orbital CT images to train a 2-dimensional U-Net for orbital tumor detection and segmentation. The U-Net, containing an encoder and a decoder, has a U-shaped structure. During the encoding stage, multi-scale feature maps are extracted from input images through multiple down-sampling. The size of the feature map decreased from 256 × 256 as input to 16 × 16 at the bottom, and the number of channels increased from 1 at the origin to 1024 at the last. Inversely, in the decoding stage, the up-sampling operation was conducted to sequentially recover high-level semantic information. Meanwhile, the low-level details of different scales at the down-sampling step were combined with the high-level semantics at the up-sampling step by skip connection, which was conducive to generating more accurate segmentation masks. The size of the feature map increased from 16 × 16 to 256 × 256, and the number of channels decreased from 1024 to 1. The output was a probability map of the orbital tumor. This probability map was binarized to get the mask images of orbital tumors, and the threshold was set to 0.5. The local tiny outliers were eliminated using a denoising approach to further enhance the segmentation performance. Additionally, the small cavities within the segmented orbital tumors were fulfilled by the morphological process. The final boundary of the segmented orbital tumor was smoothed using median filtering to approximate the shape of ground truth. Gradient descent was performed using the Adam optimizer, and cross entropy was employed to represent the loss function. The batch size was set to 2, and the epoch was set to 300. The initial learning rate was set to 0.001; then, every 80 epochs it was reduced by a factor of 10.

#### 2.3.3. Classification Network

The classification network was based on ResNet-34 [34], which used 3 × 3 convolution kernels to extract high-level features of CT images through the deep structure. It was composed of an input convolutional layer, a max-pooling layer, 16 residual blocks, an average pooling layer, a fully connected layer, and a softmax layer. Each residual block consisted of two convolution layers and an identity mapping. Three of these residual blocks were special. Since the convolution layer changed the size of the feature map and the number of channels, the identity mapping additionally introduced the convolution kernel of 1 × 1 to reduce the size of the feature map and increase the number of channels. It was performed with a stride of 2. The identity mapping structure of ResNet-34 avoided the problem of gradient vanishing during gradient descent and preserved the network’s deep hierarchical structure at the same time, which enabled the network to extract the higher-order image features. This guaranteed the accuracy of prediction. The final output was changed from 1000 to 2 classes to identify malignant and benign tumors. Gradient descent was performed using the Adam optimizer, and cross entropy was employed to represent the loss function. The batch size was set to 1 and the epoch to 50. The initial learning rate was set to 0.01; then, every 20 epochs, it was reduced by a factor of 10.

The proposed method was trained on a machine with NVIDIA GeForce GTX970 on an Intel i7-4770K CPU with 16 GB RAM.

### 2.4. Assessment by Ophthalmologists

To compare the classification performance between the proposed method and clinicians’ visual assessment, three ophthalmologists independently reviewed all the axial CT images in the testing set. The images were anonymized before visual analysis. All ophthalmologists were blinded to the histopathological results, and the diagnosis was made based on image features including the location, number, homogeneity, and boundaries of the lesions. Then, the sensitivity, specificity, and diagnostic accuracy of the visual assessment were calculated according to the histopathological results.

### 2.5. Statistical Analysis

The segmentation performance of orbit mass was quantitatively assessed using dice similarity of coefficient (DSC) [35] by comparison of manual annotations and automatic segmentations. The DSC is defined as

DSC = 2 × TP / (FP + 2 × TP + FN) (1)

where TP, FP, and FN are the numbers of true positive, false positive, and false negative detections, respectively. To visualize the automatic segmentation results, the recall-to-dice curve and the precision-to-recall curve were also drawn. Precision and recall are defined as

Precision = TP / (TP + FP) (2)

Recall = TP / (TP + FN)(3)

The classification performance based on DL was evaluated by the receiver operating characteristics curve (ROC) and the area under the ROC (AUC). In addition, the sensitivity, specificity, accuracy, false-positive rate, false-negative rate, positive predictive rate, and negative predictive rate of the automatic classification model and 3 ophthalmologists were calculated to further evaluate the diagnosis ability of the proposed automatic orbital tumor system.

Sensitivity = TP / (TP + FN)(4)

Specificity = TN / (TN + FP)(5)

Accuracy = (TP + TN) / (TP + TN+ FP + FN)(6)

False-positive rate = FP / (FP + TN)(7)

False-negative rate = FN / (TP + FN)(8)

Positive predictive rate = TP / (TP + FP)(9)

Negative predictive rate = TN / (TN + FN)(10)

where TP, TN, FP, and FN are the numbers of true positive, true negative, false positive, and false negative classifications.

Independent samples *t*-test and Chi-square test were used to analyze the differences in age and sex, respectively. The diagnostic performance of the DL-based system and 3 ophthalmologists were evaluated using the Chi-square test. All the above statistical analyses were performed using SPSS Statistics 20.0 (IBM Corporation, Armonk, NY, USA). A *p* value less than 0.05 was considered statistically significant. The DSC was calculated using MATLAB R2015b (The MathWorks, Natick, MA, USA). The ROC and confusion matrices were obtained with Python 3.6 using the Matplotlib package.

## 3. Results

### 3.1. Demographic Data

A total of 64 patients (mean age: 51.69 years old), including 36 males and 28 females were recruited in this study. The benign orbital tumor group consisted of 35 patients (mean age: 47.46 years old), and the malignant orbital tumor group included 29 patients (mean age: 56.79 years old). The demographic information is shown in Table 1. There were no significant differences between these two groups in terms of age and sex. Histopathological exam results showed that all patients had cavernous hemangioma in the benign orbital tumor group. In the malignant group, 25 patients (86.21%) had mucosa-associated lymphoid tissue (MLAT), 3 patients (10.34%) had diffuse large B-cell lymphoma (DLBL), and 1 patient (3.45%) had follicular lymphoma (FL).

### 3.2. Tumor Segmentation in CT images

**Figure 2:**
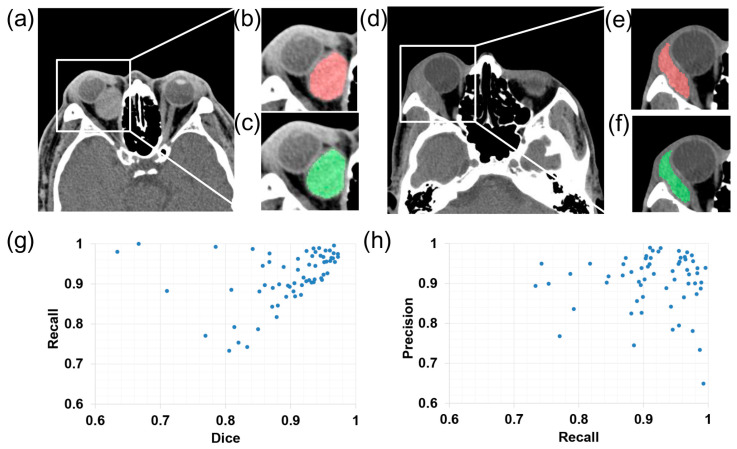
Segmentation performance of the deep learning algorithm: (**a**,**d**) representative original computed tomography images from patients with hemangioma and lymphoma, respectively; (**b**,**e**) manual annotations which are shown in red; (**c**,**f**) automatic segmentation results which are shown in green; (**g**,**h**) recall-to-dice and precision-to-recall scatter diagrams.

A total of 69 axial CT images were randomly selected as the testing set, and this testing set was used in both segmentation and classification networks. Figure 2 shows the representative segmentation results of the benign and malignant orbital tumors using the proposed DL algorithm. Figure 2a,d show the original non-contrast-enhanced CT images of the patients with cavernous hemangioma and lymphoma, respectively. Comparison of the ground truth [red annotations in Figure 2b,e] and DL algorithm predicted segmentation [green annotations in Figure 2c,f] showed good performance in segmenting orbital tumors of different natures. The DSC quantitively described the overlap of the ground truth and the predicted result. The mean DSC value was 0.89 in the testing set. The recall-to-dice and precision-to-recall scatter diagrams in Figure 2g,h display the detailed segmentation results of all images in the testing set, demonstrating that high precision and recall were achieved in most images.

### 3.3. Tumor Classification

**Figure 3:**
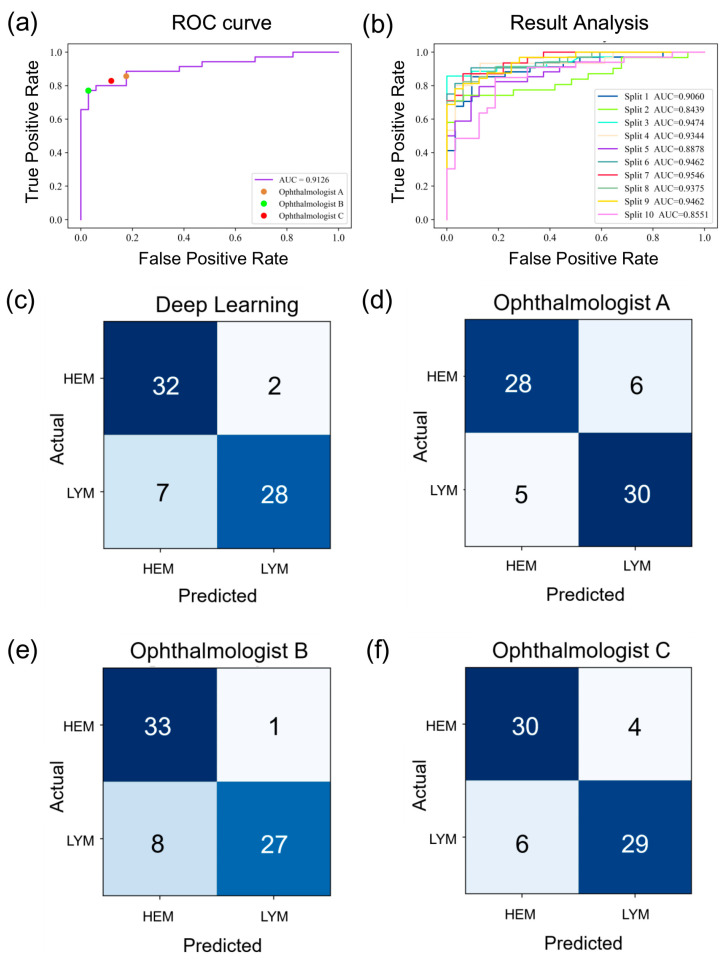
Classification performance of deep-learning based model and 3 ophthalmologists: (**a**) the receiver operating characteristic (ROC) curve of ResNet-34; (**b**) result analysis of the 10-fold cross-validation; (**c**–**f**) confusion matrices of the deep-learning-based model and 3 ophthalmologists.

The ROC analysis demonstrates the effectiveness of ResNet-34 in benign and malignant orbital tumor classification, with an AUC of 0.9126. The diagnostic results of three ophthalmologists are also visualized in Figure 3a. Since the dataset used in this study was relatively small and from two individual institutions, 10-fold cross-validation was carried out to verify the stability and robustness of the proposed DL methods. The entire dataset of 602 images was randomly divided into 10 folds. At each passage, nine folds were randomly taken as the training set, and the remaining fold was taken as the testing set. This process was repeated 10 times to make sure each image had been trained and tested. Figure 3b shows that a consistent performance was obtained, with the AUC of the binary classification ranging from 0.8439 to 0.9546. Figure 3c–f show the confusion matrices of the DL-based model and three ophthalmologists, respectively. The number of orbital tumors that were misdiagnosed by the DL algorithm and ophthalmologist B was the same (nine images, 13.04%), which was smaller than the other ophthalmologists. There are 11 (15.94%) and 10 (14.49%) images that were misclassified by ophthalmologists A and C, respectively.

### 3.4. Comparison with Ophthalmologists’ Assessment

**Figure 4:**
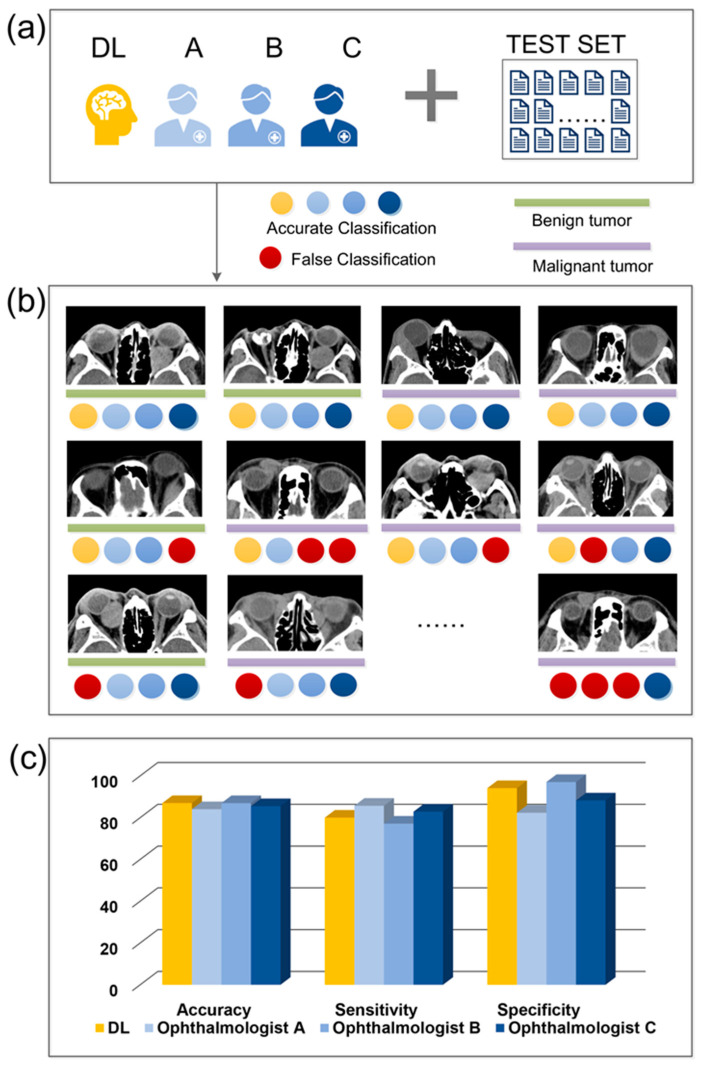
(**a**) Comparison of classification performance between the DL-based model and 3 ophthalmologists in the test set; (**b**) representative classification results; (**c**) comparison of the accuracy, sensitivity, and specificity of DL-based model and 3 ophthalmologists.

The Chi-square test showed no significant difference between the diagnostic performance of the DL-based system and three ophthalmologists (*p* > 0.05). Figure 4b displays representative examples of tumor classification. Table 2 shows that the accuracy of ResNet-34 was equal to ophthalmologist B (86.96%) and higher than ophthalmologist A (84.06%) and C (85.51%). The sensitivity of the DL-based model and three ophthalmologists was 80.00%, 85.71%, 77.14%, and 82.86%. The specificity was 94.12%, 82.35%, 97.06%, and 88.24%, respectively. Compared with ophthalmologist B, the false-negative rate of the DL algorithm was lower (20.00% versus 22.86%).

## 4. Discussion

In this retrospective study, we established a multicenter dataset consisting of patients with cavernous hemangioma and lymphoma and proposed an end-to-end DL-based system to automatically segment and classify benign and malignant orbital tumors. The diagnostic performance of this automatic system was further explored by comparison with three certified ophthalmologists. The results showed that the automatic orbital tumor system could accurately segment orbital tumors on non-contrast-enhanced CT images with a mean DSC of 0.89. The diagnostic performance of the DL-based system was equal to three ophthalmologists.

Early recognition and diagnosis of orbital tumors are critical for treatment decisions and follow-up management. The discovery of benign tumors in orbit does not necessarily mandate its invasive treatment. A proportion of patients with asymptomatic benign orbital tumors may never require excision. Scheuerle et al. reported that all orbital hemangioma patients who received conservative treatment remained stable with a follow-up period between 3 to 10 years [3]. Patients with malignant orbital tumors may receive more aggressive treatment options, including surgery, radiotherapy, and chemotherapy [6]. A closer follow-up is also required to monitor the progress of malignant orbital tumors and ensure early intervention when recurrence is detected. Additionally, the advanced clinical stage of malignant tumors was proved to be the variable that was significantly associated with poor prognosis [36,37]. Hence, early detection of orbital tumors is also of critical importance for the prediction of prognosis.

It is often considered essential to perform a surgical orbital biopsy to confirm the diagnosis of orbital masses with unknown aetiology [38]. The surgical orbital biopsy could simultaneously be the diagnostic and therapeutic procedure for patients who need surgical excision due to visual threat or suspicion of malignancy. Nevertheless, for patients with benign orbital tumors that can be managed conservatively, this invasive procedure could merely be used as a diagnostic criterion. In these cases, the accuracy of non-invasive or minimally invasive diagnostic modalities should be improved.

CT and MRI, exhibiting the location, content, soft tissue, and bone characteristics of orbital tumors, are important tools to assist ophthalmologists in making the preliminary diagnosis. However, the accuracy of clinical and radiological diagnosis was not always satisfactory, and the positive prediction rate relied highly on the skills and knowledge of the reviewers. Koukkoulli et al. [39] retrospectively analyzed patients who underwent surgical orbital biopsy from 2003 to 2015, involving more than 100 orbital lesions. The accuracy of the diagnoses made by the ophthalmologists and the radiologists was compared with histological results. The results showed that correct diagnoses were reached in less than 50% of all cases reviewed by ophthalmologists and radiologists alike. In addition, 34.8% and 39.3% of all cases reviewed by ophthalmologists and radiologists reported no differential diagnosis. In practical clinical settings, ophthalmologists often asked a detailed history and performed a comprehensive ocular examination, and then correctly concluded that orbital imaging was required but reached no specific diagnosis. Radiologists often reported the imaging findings using suggestive language and provided a list of possible diagnoses. In our study, the accuracy of differentiating benign and malignant orbital tumors reached 86.96%, exhibiting the robustness of the proposed system. The AUC of 10-fold cross-validation ranged from 0.8439 to 0.9546, which indicated the stability and repeatability of this DL-based system.

To differentiate benign and malignant orbital tumors, several studies attempted to interpret CT and/or MRI to figure out predictive features, but the results were not always consistent. Ben et al. [17] established a guideline for reviewing orbital CT and/or MRI based on 84 features. Applying this guideline, three physicians reevaluated all images. Then, the differential ability of the multiple features to discriminate between benign, malignant, and inflammatory orbital lesions was calculated. None of these features had the high sensitivity to distinguish the nature of different orbital lesions. Xian et al. [10] reported that some MRI image features were associated with malignant orbital lesions, and the inter-observer agreement between the two observers was excellent. The features that had a high sensitivity and specificity for the prediction of malignant lesions were the involvement of preseptal space, ill-defined margin, molding around orbital structures, isointensity on T2-weighted MRI images, homogeneous enhancement, and washout-type time-intensity curve (TIC). Yuan et al. [40] further explored the diagnostic efficacy of TIC on dynamic contrast-enhanced MRI images. The results demonstrated that the orbital mass with a washout pattern of TIC may be more likely to be malignant, while those having the persistent pattern of TIC would be more suggestive to be identified as benign. In addition, Khalek et al. [41] found that the apparent diffusion coefficient value at three-T diffusion MRI was a feature that could diagnose malignant versus benign orbital lesions.

The aforementioned studies indicated that features of benign and malignant orbital masses may be overlapped, and it may be difficult to establish an efficient feature map. Furthermore, the detection and annotation of orbital lesions were still manual. Some features, such as the shape and margin of masses, were assessed by ophthalmologists or radiologists. Such processes with human involvement may be influenced by the experience of the reviewer. The lack of ophthalmologists [42] and the increased workload of radiologists [43] make it even harder to accurate diagnosis of benign and malignant orbital tumors. In this study, the proposed end-to-end orbital tumor diagnosis system applied the algorithm to automatically analyze the high-level features of non-contrast-enhanced CT, discerning malignant from benign tumors without any manual processing.

AI-based models could help improve the precision of image interpretation. Several studies had showed the efficiency of AI-based algorithms in distinguishing orbital masses on CT and MRI. Guo et al. [27] employed an MR-based radiomics signature to distinguish ocular adnexal lymphoma (OAL) and idiopathic orbital inflammation (IOI), which reached an AUC of 0.74 and 0.73 in primary and validation cohorts. Han et al. [44] used an ML-based model to automatically identify the differences in orbital cavernous venous malformation from non-contrast-enhanced CT. Hou et al. [28] reported a bag-of-features (BOF)-based radiomic analysis method, with a support vector machine as the classifier. Differentiation with augmentation achieved an AUC of 0.803 in the test group from contrast-enhanced MRI, but the same analysis results from pre-contrast MRI were significantly less reliable. Radiomics has the advantage of extracting and analyzing high-dimensional quantitative features obtained from medical images at high throughput [45]. However, the efficacy of radiomics-based methods relied on the segmentation accuracy of the ROI, the quantity and type of extracted features, and the choice of the classifier. In the studies mentioned above, ROI segmentation was conducted manually, which may be affected by the subjective bias of observers. Bi et al. [46] applied three DL models to differentiate cavernous hemangiomas from schwannomas. The first two models were employed for eye and tumor location. The third classification model had an accuracy of 91.13% in the transverse T1 CE sequence. However, the highest accuracies were achieved in CE MRI, an invasive imaging examination. In this study, we simplified the workflow. In the testing set, after inputting non-contrast-enhanced CT images, the DL-based system could segment the tumoral regions and output the diagnosis of benign and malignant orbital tumors end-to-end, reducing the workload of clinicians.

Although the present study demonstrated the potential utility of DL in the detection and classification of orbital tumors, it had several limitations. First, the number of patients included was relatively small. For further studies, a larger number of cases and more types of orbital tumors are necessary. Second, in this retrospective study, the CT images selected had different slice thicknesses and scanning parameters with different CT scanners. However, these differences of input data also demonstrated the generalizability of the proposed method. Third, although the diagnosis performance reached a satisfactory result that was equal to three certified ophthalmologists, it was not compared with contrast-enhanced CT or MRI images. Therefore, it is promising to use different kinds of input images and make comparisons between CT and MRI images, as well as between non-contrast-enhanced and contrast-enhanced images. Fourth, the axial CT scans used in this study could only provide two-dimensional tumor features. In the future, location features should be combined with three-dimensional medical images to improve the diagnosis performance of automatic orbital tumor analysis systems.

## 5. Conclusions

In conclusion, the results of our study showed that the proposed DL-based system could effectively segment and classify different orbital tumors. It may provide accurate diagnostic assistance for inexperienced clinicians and help make treatment decisions. In the future, with the improvement of DL technology, these techniques have the potential to assist in the diagnosis of different tumors in several bodily areas.

## Figures and Tables

**Table 1:** Demographic data of patients with orbital tumors.

Items	Total	Hemangioma	Lymphoma
Number of patients	64	35	29
Male	36	17	19
Female	28	18	10
Mean age (years)	51.69	47.46	56.79

**Table 2:** Diagnostic performance of ResNet-34 and 3 ophthalmologists.

Items	ResNet-34	Ophthalmologist		
		A	B	C
Sensitivity (%)	80.00	85.71	77.14	82.86
Specificity (%)	94.12	82.35	97.06	88.24
False-Positive Rate (%)	5.88	17.65	2.94	11.76
False-Negative Rate (%)	20.00	14.29	22.86	17.14
Positive Predictive Value (%)	93.33	83.33	96.43	87.88
Negative Predictive Value (%)	82.05	84.85	80.49	83.33
Accuracy (%)	86.96	84.06	86.96	85.51
AUC	0.9126	/	/	/

## Data Availability

The data presented in this study are available on request from the corresponding author. The data are not publicly available due to privacy issue.

## References

[1] Shields J.A., Shields C.L., Scartozzi R. (2004). Survey of 1264 patients with orbital tumors and simulating lesions—The 2002 montgomery lecture, part 1. Ophthalmology.

[2] Rootman D.B., Heran M.K., Rootman J., White V.A., Luemsamran P., Yucel Y.H. (2014). Cavernous venous malformations of the orbit (so-called cavernous haemangioma): A comprehensive evaluation of their clinical, imaging and histologic nature. Br. J. Ophthalmol..

[3] Scheuerle A.F., Steiner H.H., Kolling G., Kunze S., Aschoff A. (2004). Treatment and long-term outcome of patients with orbital cavernomas. Am. J. Ophthalmol..

[4] Harris G.J. (2010). Cavernous hemangioma of the orbital apex: Pathogenetic considerations in surgical management. Am. J. Ophthalmol..

[5] Low C.M., Stokken J.K. (2021). Typical orbital pathologies: Hemangioma. J. Neurol. Surg. B Skull Base.

[6] Woolf D.K., Ahmed M., Plowman P.N. (2012). Primary lymphoma of the ocular adnexa (orbital lymphoma) and primary intraocular lymphoma. Clin. Oncol..

[7] Kharod S.M., Herman M.P., Morris C.G., Lightsey J., Mendenhall W.M., Mendenhall N.P. (2018). Radiotherapy in the management of orbital lymphoma a single institution’s experience over 4 decades. Am. J. Clin. Oncol.-Cancer Clin. Trials.

[8] Demirci H., Shields C.L., Shields J.A., Honavar S.G., Mercado G.J., Tovilla J.C. (2002). Orbital tumors in the older adult population. Ophthalmology.

[9] Khan S.N., Sepahdari A.R. (2012). Orbital masses: CT and MRI of common vascular lesions, benign tumors, and malignancies. Saudi. J. Ophthalmol..

[10] Xian J.F., Zhang Z.Y., Wang Z.C., Li J., Yang B.T., Man F.Y., Chang Q.L., Zhang Y.T. (2010). Value of MR imaging in the differentiation of benign and malignant orbital tumors in adults. Eur. Radiol..

[11] Zhang L., Li X., Tang F., Gan L., Wei X. (2020). Diagnostic imaging methods and comparative analysis of orbital cavernous hemangioma. Front. Oncol..

[12] Russell E.J., Czervionke L., Huckman M., Daniels D., McLachlan D. (1985). Ct of the inferomedial orbit and the lacrimal drainage apparatus: Normal and pathologic anatomy. AJR Am. J. Roentgenol..

[13] Langer B.G., Mafee M.F., Pollack S., Spigos D.G., Gyi B. (1987). Mri of the normal orbit and optic pathway. Radiol. Clin. N. Am..

[14] Joseph A.K., Guerin J.B., Eckel L.J., Dalvin L.A., Keating G.F., Liebo G.B., Benson J.C., Brinjikji W., Laack N.N., Silvera V.M. (2022). Imaging findings of pediatric orbital masses and tumor mimics. Radiographics.

[15] Priego G., Majos C., Climent F., Muntane A. (2012). Orbital lymphoma: Imaging features and differential diagnosis. Insights. Imaging.

[16] Beckett K.R., Moriarity A.K., Langer J.M. (2015). Safe use of contrast media: What the radiologist needs to know. Radiographics.

[17] Ben Simon G.J., Annunziata C.C., Fink J., Villablanca P., McCann J.D., Goldberg R.A. (2005). Rethinking orbital imaging—Establishing guidelines for interpreting orbital imaging studies and evaluating their predictive value in patients with orbital tumors. Ophthalmology.

[18] Nichols J.A., Herbert Chan H.W., Baker M.A.B. (2019). Machine learning: Applications of artificial intelligence to imaging and diagnosis. Biophys. Rev..

[19] Cho S.M., Austin P.C., Ross H.J., Abdel-Qadir H., Chicco D., Tomlinson G., Taheri C., Foroutan F., Lawler P.R., Billia F. (2021). Machine learning compared with conventional statistical models for predicting myocardial infarction readmission and mortality: A systematic review. Can. J. Cardiol..

[20] Brehar R., Mitrea D.A., Vancea F., Marita T., Nedevschi S., Lupsor-Platon M., Rotaru M., Badea R.I. (2020). Comparison of deep-learning and conventional machine-learning methods for the automatic recognition of the hepatocellular carcinoma areas from ultrasound images. Sensors.

[21] Ye Y., Xiong Y., Zhou Q., Wu J., Li X., Xiao X. (2020). Comparison of machine learning methods and conventional logistic regressions for predicting gestational diabetes using routine clinical data: A retrospective cohort study. J. Diabetes. Res..

[22] Ting D.S.W., Cheung C.Y.L., Lim G., Tan G.S.W., Quang N.D., Gan A., Hamzah H., Garcia-Franco R., San Yeo I.Y., Lee S.Y. (2017). Development and validation of a deep learning system for diabetic retinopathy and related eye diseases using retinal images from multiethnic populations with diabetes. JAMA.

[23] Ting D.S.W., Pasquale L.R., Peng L., Campbell J.P., Lee A.Y., Raman R., Tan G.S.W., Schmetterer L., Keane P.A., Wong T.Y. (2019). Artificial intelligence and deep learning in ophthalmology. Br. J. Ophthalmol..

[24] Bao X.-L., Sun Y.-J., Zhan X., Li G.-Y. (2022). Orbital and eyelid diseases: The next breakthrough in artificial intelligence?. Front. Cell. Dev. Biol..

[25] Pan L., Chen K., Zheng Z., Zhao Y., Yang P., Li Z., Wu S. (2022). Aging of chinese bony orbit: Automatic calculation based on UNet++ and connected component analysis. Surg. Radiol. Anat..

[26] Li Z., Chen K., Yang J., Pan L., Wang Z., Yang P., Wu S., Li J. (2022). Deep learning-based ct radiomics for feature representation and analysis of aging characteristics of asian bony orbit. J. Craniofac. Surg..

[27] Guo J., Liu Z., Shen C., Li Z., Yan F., Tian J., Xian J. (2018). Mr-based radiomics signature in differentiating ocular adnexal lymphoma from idiopathic orbital inflammation. Eur. Radiol..

[28] Hou Y., Xie X., Chen J., Lv P., Jiang S., He X., Yang L., Zhao F. (2021). Bag-of-features-based radiomics for differentiation of ocular adnexal lymphoma and idiopathic orbital inflammation from contrast-enhanced mri. Eur. Radiol..

[29] Hua K.-L., Hsu C.-H., Hidayati H.C., Cheng W.-H., Chen Y.-J. (2015). Computer-aided classification of lung nodules on computed tomography images via deep learning technique. Onco Targets Ther..

[30] Nam J.G., Park S., Hwang E.J., Lee J.H., Jin K.-N., Lim K.Y., Vu T.H., Sohn J.H., Hwang S., Goo J.M. (2019). Development and validation of deep learning-based automatic detection algorithm for malignant pulmonary nodules on chest radiographs. Radiology.

[31] Gao X.W., Hui R., Tian Z. (2017). Classification of ct brain images based on deep learning networks. Comput. Methods Programs Biomed..

[32] Balkenende L., Teuwen J., Mann R.M. (2022). Application of deep learning in breast cancer imaging. Semin. Nucl. Med..

[33] Yin X.X., Sun L., Fu Y., Lu R., Zhang Y. (2022). U-net-based medical image segmentation. J. Healthc. Eng..

[34] He K., Zhang X., Ren S., Sun J. Deep residual learning for image recognition. Proceedings of the 2016 IEEE Conference on Computer Vision and Pattern Recognition (CVPR).

[35] Dice L.R. (1945). Measures of the amount of ecologic association between species. Ecology.

[36] Hsu C.R., Chen Y.Y., Yao M., Wei Y.H., Hsieh Y.T., Liao S.L. (2021). Orbital and ocular adnexal lymphoma: A review of epidemiology and prognostic factors in Taiwan. Eye.

[37] Olsen T.G., Heegaard S. (2019). Orbital lymphoma. Surv. Ophthalmol..

[38] Mombaerts I., Ramberg I., Coupland S.E., Heegaard S. (2019). Diagnosis of orbital mass lesions: Clinical, radiological, and pathological recommendations. Surv. Ophthalmol..

[39] Koukkoulli A., Pilling J.D., Patatas K., El-Hindy N., Chang B., Kalantzis G. (2018). How accurate is the clinical and radiological evaluation of orbital lesions in comparison to surgical orbital biopsy?. Eye.

[40] Yuan Y., Kuai X.P., Chen X.S., Tao X.F. (2013). Assessment of dynamic contrast-enhanced magnetic resonance imaging in the differentiation of malignant from benign orbital masses. Eur. J. Radiol..

[41] Khalek A.A., Razek A., Elkhamary S., Mousa A. (2011). Differentiation between benign and malignant orbital tumors at 3-T diffusion mr-imaging. Neuroradiology.

[42] Resnikoff S., Felch W., Gauthier T.M., Spivey B. (2012). The number of ophthalmologists in practice and training worldwide: A growing gap despite more than 200,000 practitioners. Br. J. Ophthalmol..

[43] Bruls R.J.M., Kwee R.M. (2020). Workload for radiologists during on-call hours: Dramatic increase in the past 15 years. Insights Imaging.

[44] Han Q., Du L., Mo Y., Huang C., Yuan Q. (2022). Machine learning based non-enhanced ct radiomics for the identification of orbital cavernous venous malformations: An innovative tool. J. Craniofac. Surg..

[45] Lambin P., Rios-Velazquez E., Leijenaar R., Carvalho S., van Stiphout R.G., Granton P., Zegers C.M., Gillies R., Boellard R., Dekker A. (2012). Radiomics: Extracting more information from medical images using advanced feature analysis. Eur. J. Cancer..

[46] Bi S., Chen R., Zhang K., Xiang Y., Wang R., Lin H., Yang H. (2020). Differentiate cavernous hemangioma from schwannoma with artificial intelligence (AI). Ann. Transl. Med..

